# Supplementary material for: Enhancing Polysulfone Mixed-Matrix Membranes with Amine-Functionalized Graphene Oxide for Air Dehumidification and Water Treatment
Source: Membranes (Basel). 2023 Jul 19;13(7):678. doi: 10.3390/membranes13070678 (PMC10383170; doi:10.3390/membranes13070678)
Supplement: Supplementary file 1 [file membranes-13-00678-s001.zip › membranes-2429791-supplementary.pdf]

Supplementary information

Enhancing Polysulfone Mixed-Matrix Membranes with Amine-Functionalized Graphene Oxide for Air Dehumidification and Water Treatment

Omnya Abdalla [1,2], Abdul Rehman [1], Ahmed Nabeeh [1], Md A. Wahab [1], Ahmed Abdel-Wahab [1] and Ahmed Abdala [1,*]

[1] Chemical Engineering Program, Texas A&M University at Qatar, Doha 23874, Qatar; omnya.abdalla@qatar.tamu.edu or o.abdalla@gord.qa (O.A.)

[2] Gulf Organisation for Research & Development (GORD), Qatar Science & Technology Park, Tech1 Bldg, Suite 203, Doha 210162, Qatar

* Correspondence: ahmed.abdala@qatar.tamu.edu; Tel.: +974-4423-0180

Table S1: Elemental Analysis of GO and GO-NH2 Obtained from XPS

Sample	Atomic %		
	%C	%O	%N
**GO**	63.15	36.85	0.00
**GO-NH_2**	77.58	15.07	7.35

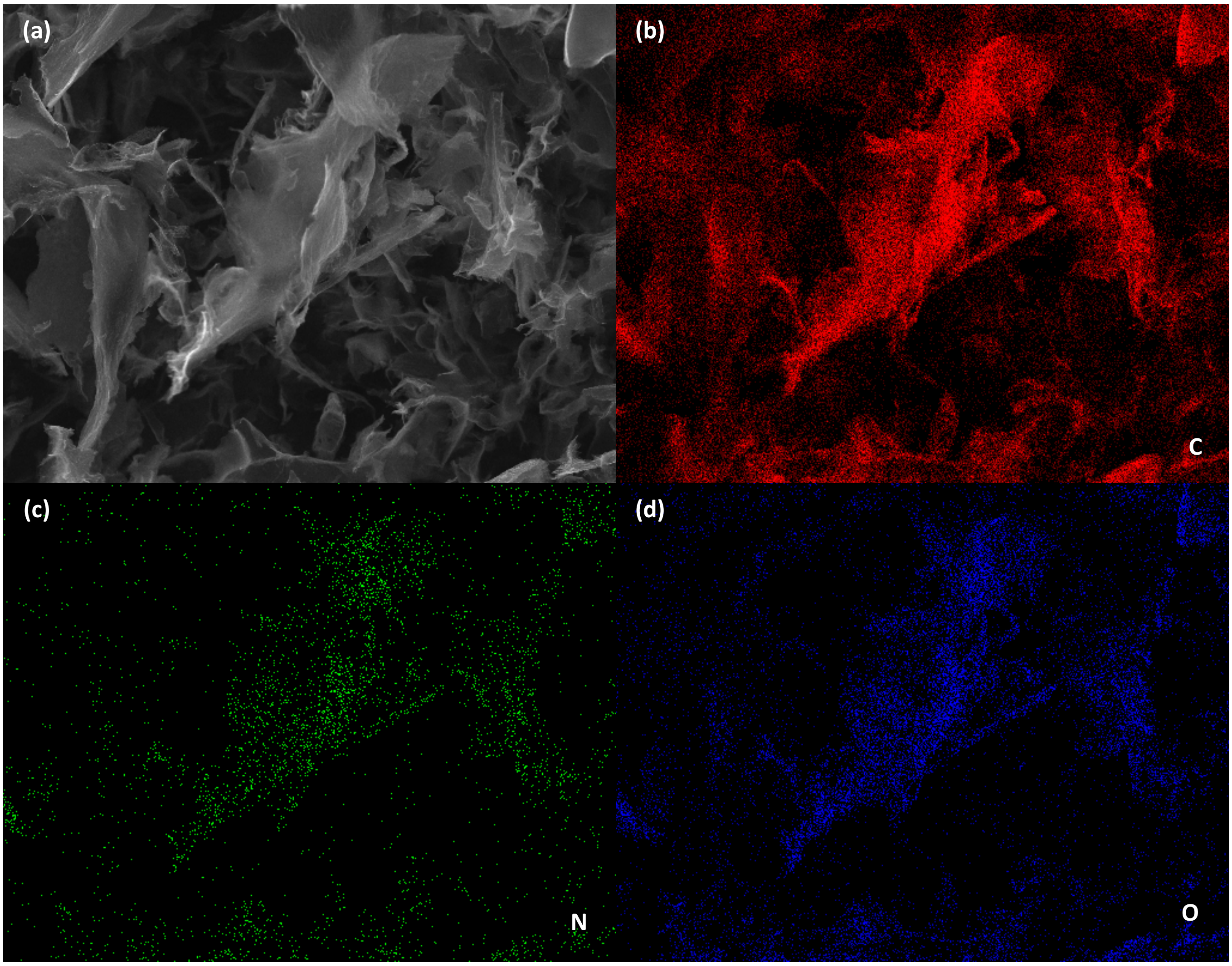

Figure S1: SEM of GO-NH_2 and EDS mapping of (b) carbon, (c) nitrogen, and (d) oxygen atoms of GO-NH_2.

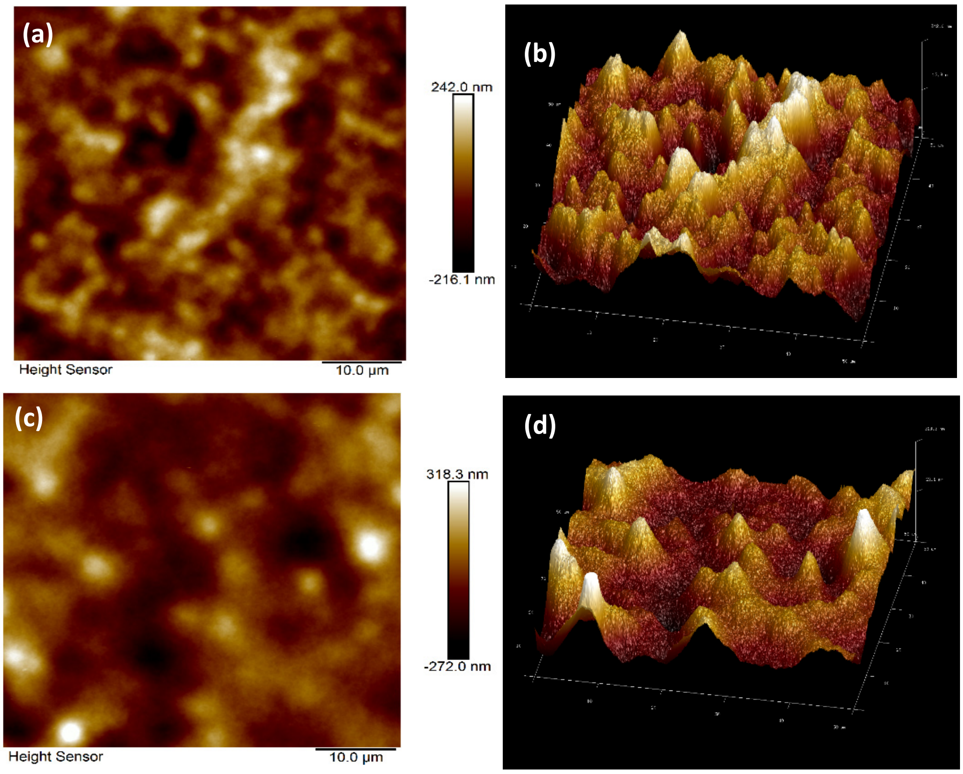

Figure S2: Surface Topography Analysis of GO-NH_2 Membranes: (a) PSf, (b) Roughness of PSf, (c) Surface of PSf-0.1-GO-NH_2, and (d) Roughness of PSf-0.1-GO-NH_2.
